# Editorials

**Published:** 1910-05

**Authors:** 


### EDITORIALS

**The Business Office of the JOURNAL-RECORD is 1 1-2 to 5 1-2 South Broad Street.**
**The Editorial Office is 1014-15 Century Building.**
**Address all Business Communications to *Journal-Record of Medicine*, 1 1-2 to 5 1-2 South Broad Street.**
**Make remittances payable to *THE JOURNAL-RECORD OF MEDICINE.***
**On matters pertaining to the Editorial and Original Communications, address Edgar G. Ballenger, M. D., Atlanta, Ga.**
**Reprints of Original Articles will be furnished at cost price. Requests for the same should always be made in THE MANUSCRIPT.**
**We will present, postpaid, on request, to each contributor of an original article, twenty (20) marked copies of THE JOURNAL-RECORD OF MEDICINE containing such article.**

### MEETING OF THE GEORGIA MEDICAL ASSOCIATION.

The sixty-first annual meeting of this Association was held in Athens, April 20, 21 and 22, and while very enjoyable from a social standpoint, was so rushed for time owing to the trip to Jefferson, where a monument to Crawford W. Long was unveiled, that the time for the reading of papers was greatly curtailed. It is our opinion, however, that too many papers are always read before these meetings, and we believe a program committee should be appointed by the Association to supervise and censor the papers before allowing the program to appear, and thus limit the papers to those of importance or originality upon timely or suitable subjects, and provide free discussion by men who are prepared to discuss the particular subjects, and then by the Association at large.

While such a plan would be departing from our time-honored method, which we have now outgrown, it would make the scientific meetings well worth attending and at the same time greatly stimulate better and more carefully prepared scientific papers. We do not mean ultra-scientific work, but useful, practical, everyday subjects accurately presented as viewed from a modern, up-to-date standpoint. Free discussions would then add "ginger" to the meetings.

Papers should be submitted, say three months in advance of the meetings, and then carefully read by one or more of the members of the committee, and a report made according to an honest opinion of said paper's value. If the writer has made his paper too long, advise him to condense it. If it contains only well-known, admitted text-book facts, it is manifestly unfair to the Association and a waste of time to allow such a paper to be read. If the paper is too scientific or deals with an impractical subject not of general medical or surgical interest, it should not be read. No paper should be presented that is not written, as "rambling talks" consume too much time. Let the essayist show the Association the courtesy of carefully preparing and condensing what he has to say. Many useful papers might be read by title and published in the transactions, and better read and digested at leisure than heard in the meetings.

These are but a few suggestive random thoughts which, of course, could be greatly amplified and improved upon by the discussion and suggestions of a program committee.

The next meeting is to be held in Rome, with Dr. E. C. Davis, of Atlanta, as president, and it is our hope that some such plan may be devised to lessen the number, improve the quality of the papers read, and increase the amount of general discussion.

---

### COMMENCEMENT EXERCISES OF THE ATLANTA COLLEGE OF PHYSICIANS AND SURGEONS.

After a most successful year's work, the Atlanta College of Physicians and Surgeons graduated sixty-three young doctors, May 1st. An interesting program of music and addresses was prepared. Rev. Dunbar H. Ogden, pastor of the Central Presbyterian church, delivered the address of the evening, and Judge T. P. Westmoreland conferred the degrees upon the class. Dr. W. S. Elkin, dean of the faculty, made his annual report. The certificates of proficiency were awarded by Dr. J. S. Todd. The following men received their degrees:

B. E. Alsobrook, J. M. Anderson, C. C. Aven, J. S. Beard, C. P. Benson, C. C. Brooks, D. L. Bryson, K. W. Buell, J. C. Chandler, J. H. Coffee, H. M. Cook, R. M. Coulter, I. W. Cummings, J. E. Davis, C. H. Dobbs, H. L. Earl, A. B. Elkin,

P. H. Fitzgerald, J. H. Foster, D. W. Freeman, D. L. Garrett, F. S. Glover, G. B. Hack, D. R. Handley, D. A. Haney, C. H. Harvey, C. W. Harvey, E. B. Hatch, L. M. Hawkins, W. H. Hope, P. M. Howard, W. J. Hutchins, S. E. Jacobs, J. S. Jacoby, E. A. Johnson, Z. B. Johnston, A. B. Jones, F. G. Jones, J. S. Liebman, W. G. Lowe, H. M. Luning, C. G. McCay, W. R. McCoy, F. R. Mann, W. F. Massey, C. S. Merriam, R. L. Miller, J. C. Millsap, C. A. Neves, C. E. Pattillo, D. S. Porter, G. B. Randall, T. E. Rogers, J. L. Smith, G. F. Spearman, W. F. Tanner, J. H. Terrell, T. C. Thames, J. P. Trott, J. L. Tyre, F. L. Underwood, B. F. Williams, L. F. Wright.

---

